# Lipid alterations in hereditary peripheral neuropathies: common mechanisms in disease heterogeneity?

**DOI:** 10.1186/s13024-026-00932-6

**Published:** 2026-02-04

**Authors:** Koen Kuipers, Sam Vanherle, Kirsten Poelmans, Esther Wolfs, Jeroen Bogie, Tim Vangansewinkel

**Affiliations:** 1 Department of Immunology and Infection, Biomedical Research Institute, Hasselt University, Diepenbeek, Belgium; 2 University MS Center, Hasselt University, Hasselt, Belgium; 3 Lab for Functional Imaging & Research on Stem Cells (FIERCELab), Biomedical Research Institute, Hasselt University, Diepenbeek, Belgium; 4 Department of Neurosciences, Leuven Brain Institute (LBI), KU Leuven - University of Leuven, Leuven, Belgium; 5 Peripheral Neuropathy Research Group, Department of Biomedical Sciences, University of Antwerp, Antwerp, Belgium

**Keywords:** Peripheral neuropathy, Lipid metabolism, Schwann cells, Neurons, Demyelination, Neurodegeneration

## Abstract

While the impact of lipid alterations on central nervous system disorders is well-studied, increasing evidence indicates that lipids also play an important role in the pathology of hereditary peripheral neuropathies (HPN). It is becoming clear that Schwann cells and neurons in peripheral nerves heavily depend on lipids for membrane interactions, (sub)cellular signalling, and the formation of myelin sheaths. In support of this notion, disturbances in the level and composition of lipid classes, including phospholipids, sphingolipids and cholesterol, perturb normal functioning of peripheral nerves. Intriguingly, lipid disturbances seem to be a common denominator within the heterogeneous group of HPN, with hindrances in cholesterol and sphingolipid metabolism primarily influencing Schwann cell and neuron homeostasis, respectively. In this review, we provide an overview of lipid disturbances in various HPN with the goal of finding main commonalities between the different diseases and to identify potential novel treatment strategies.

## Introduction

The peripheral nervous system (PNS) represents a highly heterogeneous entity, composed of bundles of nerve fibres grouped together and enclosed by connective tissue layers, as well as clusters of nerve cell bodies; called ganglia, found at various places outside the central nervous system (CNS) [1]. Functionally, peripheral nerves receive sensory information via afferent fibres and transmit it to the brain for processing and interpretation. Vice versa, efferent fibres carry motor commands from the brain and spinal cord back to the muscles. Furthermore, the PNS also regulates various autonomic functions, such as heart rate, blood pressure, digestion, and respiration. Additionally, the PNS is involved in controlling developmental processes [1–3], tissue regeneration and homeostasis [2, 4, 5], and in regulating mesenchymal stem cell niches by supporting the survival and differentiation of stem cells, for example via FGF1 signalling [1, 6, 7]. All in all, the PNS ensures efficient communication with the CNS while also regulating autonomic control, tissue development, maintenance and repair.

The PNS is composed of a variety of cell types, including neurons and Schwann cells, with the majority of them originating from trunk neural crest cells during vertebrate development and neurulation [8, 9]. Different types of nerve fibres exist, which can vary in size, function, and are either unmyelinated or myelinated. Myelin is a specialized structure formed by the wrapping of Schwann cell plasma membranes around large-caliber axons [10]. It serves as an electrical insulator, allowing impulses to propagate more rapidly along nerve fibres, thereby increasing the efficiency of signal transduction. Schwann cells are the main glia in the PNS, and apart from myelination, they also provide physical and trophic support to axons [11]. In vertebrates, myelination in the PNS starts shortly after birth and is typically completed within a brief period (e.g. two years after birth in humans and four weeks after birth in rodents) [12, 13]. In contrast to big axons, small calibre axons (i.e. C-type fibres < 1 μm) are unmyelinated, but they are ensheathed by Remak cells, a special type of non-myelinating Schwann cells [14].

Peripheral neuropathies encompass a range of neurological disorders characterized by damage to the peripheral nerves. These conditions can be hereditary or acquired and may result from various factors, including trauma, infections, and autoimmune reactions [15]. Despite the multitude of underlying causes, the dysregulation of lipid metabolism within myelinating Schwann cells and neurons has been shown to be a common hallmark, compromising the integrity of the myelin sheath and axons. In this review, we will assess the current state of fundamental knowledge on lipid metabolism disturbances in both Schwann cell and neuron-related hereditary peripheral neuropathies (HPN). The main goal is to highlight shared and distinct mechanisms and their implications for the pathogenesis of these diseases.

### Lipids and their metabolism in Schwann cells and peripheral neurons

In contrast to certain organelle and plasma membranes, which have an equal amount of lipids and proteins, the myelin sheath consists of 70–85% of lipids [10]. Myelin lipids undergo continuous remodelling, of which the timely synthesis, the relative contribution of certain lipid species, and their turnover rates vary during nerve development, across the lifespan and during disease [16, 17]. Furthermore, Schwann cells actively engage in the production and recycling of lipids to uphold the integrity of the myelin sheath during injury and regeneration processes [16, 18–20]. Therefore, biosynthesis, storage, and trafficking of myelin lipids are vital processes for the development and maintenance of myelin in health and disease.

While myelin proteins support structural stability, the integrity of myelin critically depends on its lipid composition [21, 22]. Particularly, phospholipids (e.g. phosphatidylethanolamine (PE)), cholesterol, and specific glycolipids such as sphingomyelin are major constituents of myelin membranes [10]. Notably, PNS myelin was found to consist of phospholipids, cholesterol, and glycolipids in a ratio of 40%:40%:20%, contrasting with the ratio of 25%:65%:10% observed in other biological membranes [23–25]. Additionally, studies indicate that the distribution of fatty acids in myelin also differs from that of other biological membranes. While monounsaturated fatty acids, such as C18:1, and saturated very long chain fatty acids (> C22) are enriched in myelin, C16:0, C18:2, C20:4, and C22:6 fatty acid levels are reduced in myelin [26, 27]. In summary, despite originating from typical cellular plasma membrane, PNS myelin displays not only a distinctive protein composition but also a unique lipid signature.

Beyond their structural role in myelin, lipids are also implicated in diverse other biological processes in the PNS. For instance, gangliosides are known to engage in axon-glia interactions, contributing to the ensheathment of axons and maintenance of the myelin sheath [28]. Further, lipid rafts, formed by the integration of sphingolipids and cholesterol in specialized membrane nanodomains, act as platforms for various membrane proteins and signaling molecules, facilitating the stimulation of signal transduction processes, including those related to myelination pathways [29]. Finally, plasmalogen phosphatidylethanolamine species, which are highly enriched in PNS myelin, provide ROS-protective properties and are able to promote myelination through AKT phosphorylation and GSK3B activation [10, 30, 31]. In conclusion, myelin lipids seem to provide more than a structural role, and any significant diversion from its healthy composition may therefore contribute to PNS disease progression.

In addition to their role in myelin formation and Schwann cell biology, lipids are also pivotal for neuronal development and maintenance. During neurogenesis, PC biosynthesis is essential for the differentiation of neurons by promoting membrane expansion and neurite outgrowth [32]. Furthermore, mature neurons are rich in phosphatidylethanolamine (PE) species and heavily rely on them for proper mitochondrial function [33]. Additionally, the localization of cardiolipin on inner mitochondrial membranes as opposed to outer mitochondrial membranes is an essential asymmetry that aids cells in controlling the rate of mitophagy, and is especially important to regulate their metabolism [34]. Finally, neurons are highly sensitive to changes in the metabolism of various ganglioside species as well, as seen primarily in the CNS in lysosomal storage disorders [35–38]. Given the importance of lipid homeostasis in proper PNS functioning, it is not surprising that perturbations in lipid composition, quantity, and metabolism can impact the onset, progression and resolution of peripheral neuropathies, as discussed in the following sections.

### Hereditary peripheral neuropathies

Hereditary peripheral neuropathies (HPN) encompass a complex group of neurological disorders characterized by heterogeneous clinical presentations and diverse genetic causes [39]. These disorders affect sensory, motor, and/or autonomic nerve fibers, leading to axonal degeneration, demyelination, and loss of motor and sensory neurons secondary to axonal and/or Schwann cell dysfunction [40]. Phenotypic classifications of HPN depend on the degree of involvement of motor, sensory, and/or autonomic nerve fibers, resulting in the following subtypes: distal hereditary motor neuropathies (dHMN), hereditary motor and sensory neuropathies (HMSN), hereditary sensory and autonomic neuropathies (HSAN), and small fiber neuropathy [40–42]. Charcot-Marie-Tooth disease (CMT) is the most common HPN, with a global prevalence of approximately 1 in 2,500 individuals, and affects myelinated motor and sensory axons. CMT consists of several subtypes, each classified based on the pattern of inheritance, clinical characteristics, and nerve conduction studies. CMT1 and CMT4 are autosomal dominant and recessive demyelinating forms, respectively. In contrast, CMT2 represents an axonal form, displaying both autosomal dominant and recessive inheritance patterns [43, 44]. Further subclassification is based on the underlying genetic defect. For example, CMT1A and CMT1E are associated with a duplication or mutation of the *peripheral myelin protein-22* gene (*PMP22*), respectively; CMTX1 is caused by mutations in the gap junction beta-1 gene (*GJB1*) encoding connexin 32; and CMT1B is associated with mutations in the myelin protein zero gene (*MPZ*) [45, 46]. Monoallelic deletion of *PMP22* also causes a neuropathy, typically in the form of episodic motor and/or sensory deficits, called hereditary neuropathy with liability to pressure palsies (HNPP) [47]. A minority of patients exhibit a more severe phenotype involving progressive axonal degeneration and early onset, known as Déjèrine–Sottas syndrome (DSS) or CMT3 [48].

In contrast to HMSN, HSAN primarily affects sensory and autonomic neurons. HSAN1 is an autosomal dominant axonal neuropathy caused by missense mutations in the *SPTLC1* or *SPTLC2* gene encoding for two subunits of serine-palmitoyltransferase (SPT), an enzyme involved in sphingolipid biosynthesis [40, 49]. Importantly, despite having a primary cellular target, cross-talk between Schwann cells and neurons typically causes mixed pathology over time, as both cell types depend on each other for proper function during development and in homeostasis (reviewed in [50]).

Given the dependence of Schwann cells and neurons on tightly regulated lipid homeostasis, there is a clear relevance for investigating lipid metabolism alterations in the context of HPN. To date, alterations in lipid metabolism have emerged as both primary drivers and secondary consequences in the aetiology of diverse HPN, as pathological processes can disrupt myelination and thereby diminish overall lipid content. In this review, we provide an overview of the current knowledge on lipids in HPN, their role in pathophysiology, and determine if there are common pathways involved across the spectrum of these disorders.

### Lipid disturbances in demyelinating HPN: Schwann cell disease

**Fig. 1:**
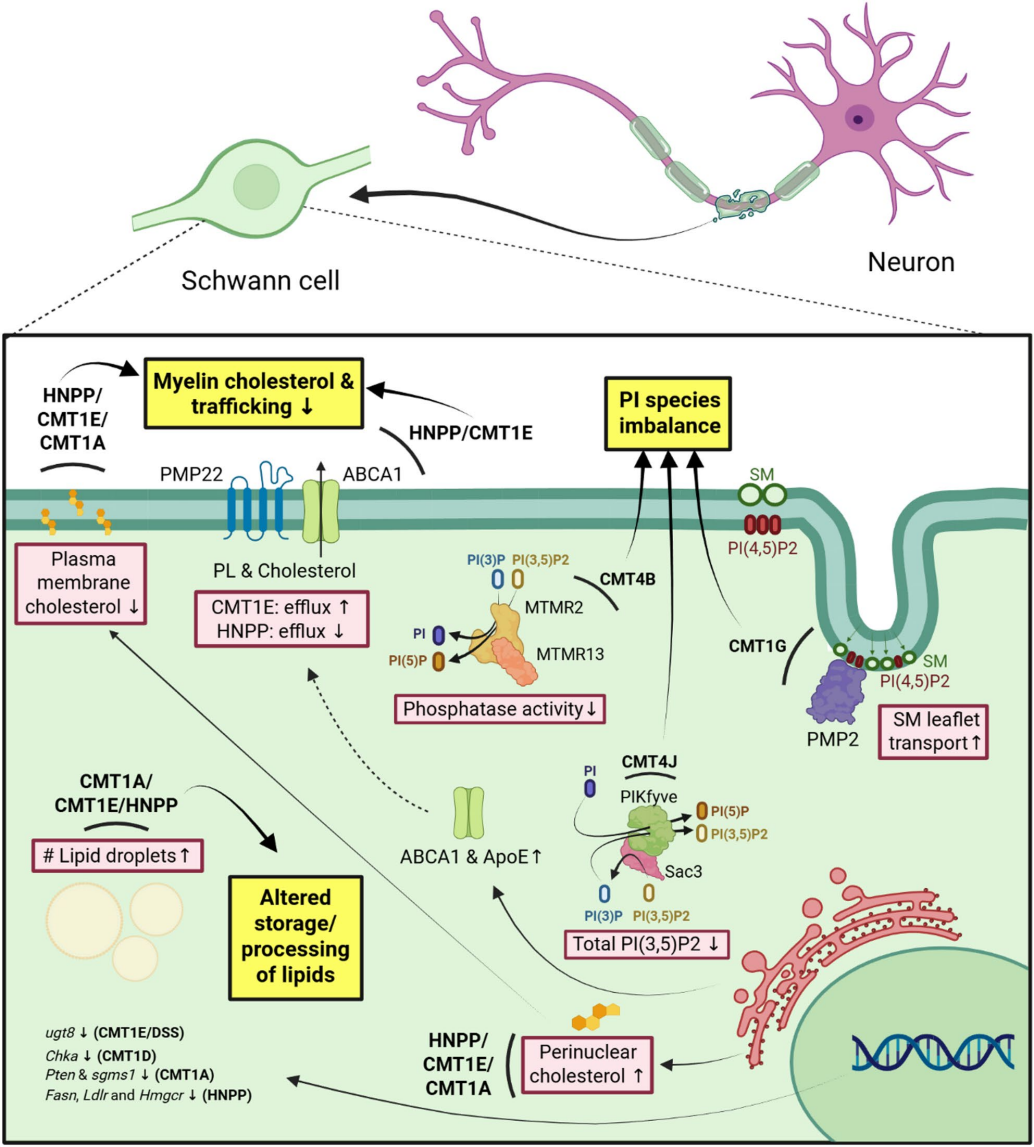
Graphical overview of common lipid-related disturbances in primary Schwann cell HPN. Altered expression of ABCA1 and cholesterol efflux are described in the pathobiology of CMT1E and HNPP. CMT1G mutations in PMP2 increase its tendency for leaflet transport of SM. CMT1A, CMT1E and HNPP Schwann cells show an increased lipid droplet load, both in PMP22-KO and TrJ sciatic nerves and after oleic acid treatment of CMT1A patient-derived iPSC-Schwann cell precursors. Perturbations in gene expression of lipid metabolism enzymes are present in HNPP, CMT1A/D/E, and DSS. CMT4B Schwann cells have a reduced phosphatase activity in MTMR2/MTMR13 complexes, leading to reduced PI/PI(5)P formation. PIKfyve-Sac3 enzyme complexes are mutated in CMT4J, resulting in a net decrease of PI(3,5)P2. Common lipid metabolism-related disturbances in Schwann cell-related HPN are depicted in yellow boxes, which includes (1) reduced cholesterol membrane content and trafficking and (2) elevated lipid droplet content in vitro and/or in vivo in all PMP22-related HPN, and (3) disturbances in PI-species due to impaired or unbalanced biosynthesis and altered membrane organization in CMT1G, CMT4B and CMT4J. HNPP: hereditary neuropathy with liability to pressure palsies; CMT: Charcot-Marie-Tooth disease; DSS: Déjèrine-Sottas syndrome; PL: phospholipid; SM: sphingomyelin; PI(P): phosphatidylinositol (phosphate); PMP22: peripheral myelin protein 22; PMP2: peripheral myelin protein 2; ABCA1: ATP-binding cassette transporter 1; ApoE: Apolipoprotein E; MTMR: myotubularin-related protein

Although demyelinating CMT phenotypes were already described in the 19th century, the molecular drivers of Schwann cell dysfunction remain poorly understood. Given the importance of lipids in myelin biogenesis and maintenance, we here provide an overview of perturbations in lipid quantity and their metabolic pathways observed in primary demyelinating forms of CMT, which have been summarized in Table 1 and Fig. 1, respectively.

#### PMP22-related Charcot-Marie-Tooth disease variants

The largest group of HPN, encompassing the autosomal dominant diseases CMT1A, CMT1E and HNPP, are all caused by alterations in the *PMP22* gene dosage or structure [51]. To date, multiple studies have investigated lipid-related disturbances in PMP22-related HPN, highlighting perturbations in lipid quantities and metabolism [52–54]. First of all, PMP22 levels and mutations have been directly linked to altered abundance and localisation of the cholesterol- and phospholipid-efflux transporter ATP-binding cassette transporter 1 (ABCA1) [55]. In *Pmp22* knockout (KO) mouse nerves, increased transcript and protein levels of Abca1 and Apolipoprotein E (ApoE) were found [55]. However, a reduction in ApoE protein levels was observed in the supernatant of *Pmp22*-KO Schwann cell cultures, correlating with lower cholesterol efflux since ApoE and cholesterol are co-secreted. In line with this observation, ABCA1 expression was found to be decreased by ~ 25% in the Schwann cell plasma membrane in the absence of PMP22 [55], suggesting that there is a co-dependent membrane translocation mechanism and that, despite upregulated gene and protein expression in *Pmp22-KO mouse nerves*, functional ABCA1-ApoE efflux is impaired. Interestingly, sciatic nerves of *Abca1-*KO mice show approximately 1.5-fold higher levels of PMP22 protein levels along with increased lipid droplet accumulations and tomacula, underscoring the bidirectional regulatory relationship between ABCA1 and PMP22. Likewise, the elevated lipid droplets and tomacula observed in Abca-1 KO nerves resemble pathological features seen in PMP22-deficiencies [55]. Additionally, nerves of Trembler J (TrJ) mice, which harbor a L16P mutation in *Pmp22* and serve as a model for CMT1E, display significant overexpression of ABCA1 and ApoE protein levels reaching 13- and 20-fold higher quantities, respectively. In TrJ nerves, ABCA1 expression is predominantly localized in Schwann cells, while ApoE is present in Schwann cells, endoneurial fibroblasts, and macrophages. The molecular mechanisms driving the bidirectional relationship between ApoE and PMP22 remain to be fully determined. However, it is known that cAMP regulates both PMP22 expression levels and ABCA1 activity through phosphorylation [56, 57]. Furthermore, PMP22 contains a cholesterol-recognizing CRAC motif and colocalizes with ABCA1, which argues for a functional relationship between both proteins in cholesterol trafficking [55, 58]. In conclusion, the abovementioned studies lead to the finding that ABCA1 changes are a common denominator in PMP22-related neuropathies.

Alongside changes in cholesterol efflux, *Pmp22*-KO mice and *Pmp22* knockdown rat Schwann cells display a marked redistribution of intracellular cholesterol, with perinuclear accumulation and decreased plasma membrane cholesterol [55]. Intriguingly, cholesterol supplementation improved myelination, internodal densities, and internodal length in ex vivo DRG cultures from heterozygous *Pmp22*-deficient mice [58]. Furthermore, TrJ mouse sciatic nerves showed perinuclear entrapment of cholesterol in the Golgi apparatus, with significantly reduced total cholesterol levels in both heterozygotes and homozygotes compared to wild-type controls [58]. These findings strongly suggest that the altered localization and/or reduction of cholesterol contributes, at least in part, to Schwann cell dysfunction in HNPP and CMT1E. Similar disturbances in cholesterol distribution were observed in CRAC-motif mutant and *PMP22* overexpressing rat Schwann cells [58]. In this context, PMP22-overexpressing mouse embryonic fibroblasts exhibited cholesterol accumulation in lysosomes [58]. Sciatic nerves of C22 and C3-PMP22 mice, two CMT1A mouse models with 7 and 4–5 human PMP22 copies respectively, as well as CMT1A-patient derived induced pluripotent stem cell Schwann cell precursors (iPSC-SCPs), demonstrated lower cholesterol levels and impaired trafficking of cholesterol to the plasma membrane, suggesting that reduced plasma membrane cholesterol levels are present in all PMP22-related HPN and are directly involved in their pathobiology rather than through a loss of myelination alone [54, 59]. Consistent with these findings, CMT1A patient-derived iPSC-SCPs contained a higher degree of membrane fluidity, visualized using the cholesterol-sensitive polarity dye Di-4-ANEPPDHQ, which correlates with reduced membrane cholesterol packing [54, 60]. Increased membrane fluidity, together with a reduction in lipid raft dynamics of CMT1A patient-derived iPSC-SCP plasma membrane, further supports the hypothesis that reduced membrane cholesterol perturbs Schwann cell membrane function [54, 61–63]. In summary, these findings indicate that CMT1E, HNPP, and CMT1A are associated with marked alterations in the handling, trafficking, and localization of cholesterol.

Apart from changes in cholesterol abundance and metabolism, transcriptomic analyses of CMT1A rat sciatic nerves revealed an overrepresentation of differentially expressed genes in biological pathways associated with not only sterol, but also glycerophospholipid, and sphingolipid anabolism and catabolism [52, 53]. Accordingly, analysis of developing CMT1A rat nerve myelin lipids demonstrated a reduced presence of sphingolipid species (mostly hexosylceramide) and long-chain sphingomyelin, which was already present at P10 and remained at P365 [16]. In addition, adult rat CMT1A PNS myelin contained significantly decreased levels of total cholesterol, PC, phosphatidylethanolamine (PE) plasmalogens, SM, cerebrosides, and divergent ceramide species, including hexosylceramides and α-galactosylceramide, when corrected for myelin protein quantity. Yet, when compared to total lipid quantities, only a decrease in PE plasmalogens and an increase in triacylglycerol (TAG) were detected in the CMT1A myelin lipid stoichiometry [52]. Interestingly, all of these alterations were counteracted after a 112-day treatment with a 3% phospholipid diet, even leading to an increase in ceramides, cerebrosides, diacylglycerol (DAG), and TAG, while also improving the interperiodic distance between myelin wraps, as well as the number of myelinated axons and neurofilament densities in CMT1A rats [52]. These findings strongly suggest that lipid abnormalities, as well as lipid availability, underpin myelin abnormalities in CMT1A. A complementary study utilizing CMT1A rat sciatic nerves further supported the presence of lipid disturbances in CMT1A, highlighting significant lipid alterations, including a decrease in long-chain fatty acids within PC, phosphatidylserine (PS), and SM, alongside higher saturation levels of fatty acids incorporated in these lipid classes. Additionally, while the abundance of phosphatidylinositol (PI), DAG, and hexosylceramides was reduced, PE (42:1) levels were markedly increased in CMT1A sciatic nerves [53]. Gene expression analysis demonstrated increased levels of *Pten* (phosphatase and tensin homolog) and *Sgms1* (sphingomyelin synthase 1) in CMT1A transgenic nerves [53]. PTEN, a phospholipid phosphatase, is known to regulate myelination, whereas SGMS1 catalyses sphingomyelin biosynthesis. Furthermore, acid sphingomyelinase activity was increased in CMT1A rat sciatic nerves, potentially contributing to the reduced abundance of SM [53]. Interestingly, in sciatic nerves from C3-PMP22 mice, a reduced abundance of numerous lipid species was observed, including cholesterol, TAG, sphingolipids, and ether and lysophospholipids (detailed in Table 1). In contrast, C3-PMP22 nerves exhibited a higher abundance of PI (42:0), ceramides, and PC species with longer chain lengths and higher degrees of saturation [54]. Notably, observations in murine CMT1A models were largely confirmed in CMT1A patient-derived iPSC-SCPs, where a notable decrease was observed in the levels of SM, sphingolipids, and phospholipids with shorter acyl chain fatty acids. Concurrently, there was an increase in shorter acyl chain fatty acids in PS and long-chain unsaturated fatty acids in PC, PI, phosphatidylglycerol (PG), PE, ether-linked PE (PE O-), and plasmalogen PE (PE P-) when compared to isogenic controls [54]. Furthermore, cell membranes of CMT1A iPSC-SCP exhibited a higher degree of fluidity, possibly reflecting the reduced abundance of saturated fatty acids [60]. Together, these findings underscore substantial lipid alterations in both peripheral nerves and Schwann cells during CMT1A pathology. However, due to the lack of direct evidence of PMP22 interaction with other lipid species described above, these changes are likely mainly due to indirect slowing or hindrance of membrane maturation and myelin sheath formation.

Alongside changes in the lipidome, a few studies found alterations in intracellular storage of lipids in PMP22-related neuropathies. Specifically, histological analysis of lipid droplets - organelles that store neutral lipids - in nerves of *Pmp22*-KO mice demonstrated their increased presence in the endoneurium (including in Schwann cells) and the perineurium [55]. Likewise, an increase in the presence of lipid droplets was observed within the endo- and perineurial spaces in sciatic nerves of TrJ mice [64]. Interestingly, a 6-week high-fat diet (HFD) in TrJ animals resulted in a marked increase in the total number of myelinated fibers, an increase in fiber diameter, and a reduction in the mean g-ratio in sciatic and phrenic nerves, with strongest effects observed in smaller nerve fibers. Together, these results indicate that an HFD enhances myelination in both sensory and motor nerves, despite the basal accumulation of neutral lipids already present in TrJ nerves [64]. In line with these findings, CMT1A patient-derived iPSC-SCPs exhibited an increased number of lipid droplets per cell following oleic acid stimulation [54]. While the precise molecular mechanisms underlying the heightened formation of lipid droplets, as well as the functional implications of increased lipid droplet load, remain unclear in PMP22-related HPN, they underscore disturbances in cellular lipid storage and a delay in lipid turnover.

Finally, aside from the TrJ mouse model, earlier studies on the Schwann cell pathophysiology in CMT1E and Déjèrine-Sottas syndrome have been conducted using the Trembler (Tr) mouse model. Primary Schwann cells from Tr mice that carry a G150A *Pmp22* mutation in the transmembrane domain 4 had a lower PC and higher PI content compared to wild-type controls in vitro [65]. Additionally, a lower total polar lipid content was observed in the sciatic nerve of Tr mice compared to wild-type mice from postnatal day 5 (P5) onwards, with the difference in absolute lipid content progressively increasing over time, peaking at P60. Interestingly, within these nerves, the relative quantity of PC and SM, when compared to total polar lipids, decreased over time between P3-P60 in healthy nerves, but stayed relatively constant in Tr mouse nerves. However, at all ages, the percentage of total PC, PI, and Cardiolipin compared to total polar lipid content was higher in Tr mouse nerves [66]. Although contradicting the results on PC found in isolated Schwann cells, these findings highlight possible differences between whole nerve and Schwann cell-specific lipid alterations in PMP22-related diseases, as well as discrepancies between actively myelinating and non-myelinating Schwann cells [65, 66]. Furthermore, fatty acid synthetase activity was strongly reduced in Tr sciatic nerves, leading to lower palmitate formation (C16:0) [67]. Additionally, Tr mouse sciatic nerves had a lower expression of ceramide galactosyltransferase, especially in the first two weeks after birth (active myelination phase) [68]. Functional lipid metabolic experiments indicated that Tr-mouse Schwann cells use acetate and ketone bodies more for phospholipid and TG biosynthesis, but less for free fatty acid and sterol production [69]. The usage of acetate within the endoneurium was further investigated over time. Tr mice younger than 1 week incorporated less acetate into phospholipids and cerebrosides, but more into cholesterol than wild-type controls. However, after 9 and 20 days of age, acetate was incorporated less into cholesterol and more into phospholipids and cerebrosides than in the wild-type groups, respectively [70]. Furthermore, Tr mice used acetate more in the biosynthesis of TG and cholesteryl esters. Although between ages P3-P50, the acetate incorporation in free fatty acids increased in normal nerves, this trend could not be found in Tr mice. Summarized, these Tr mouse studies support the idea that developmental disturbances are, at least in part, caused by secondary disturbances in sterol and lipid biosynthesis in PMP22-related diseases.

In conclusion, PMP22-related HPN seem to be at least partially caused by ABCA1-mediated cholesterol disturbances, and as a consequence, have perturbations in lipid species content, an overall reduction in myelin lipids, and disturbed lipid turnover.

#### Other Schwann cell-related Charcot-Marie-Tooth disease variants

##### CMT1G

In addition to *PMP22*-related forms of CMT, various gene mutations have been identified to cause demyelinating forms of CMT. An example is CMT1G, which is caused by autosomal dominant mutations in *peripheral myelin protein 2* (*PMP2*). Interestingly, contrary to PMP22-related neuropathies, PMP2-KO mice do not seem to harbour significant developmental peripheral nerve defects [71]. However, when peripheral nerve damage was induced or upon selective tamoxifen-induced diphtheria toxin PMP2 positive Schwann cell ablation, remyelination of motor neurons was hampered leading to a motor neuropathy phenotype and thus confirming the importance of PMP2 in myelination of a subtype of larger diameter motor axons [72, 73]. Investigations into its mode of action revealed that PMP2 enhances glutamine uptake in substrate-limited culture conditions, increases fatty acid uptake and regulates ATP production in Schwann cells [74–76]. Furthermore, PMP2 exerts its effect through interactions with integral membrane components such as PI(4,5)P2, PI(3,5)P2, and SM, which are critical for intracellular signalling [74, 77, 78]. Specifically, PMP2 has been observed to retain SM in the outer plasma membrane leaflet and PI(4,5)P2 in the inner plasma membrane leaflet [10, 74]. The pathogenic PMP2 mutation I43N enhances its affinity for binding to PI(4,5)P2, resulting in the sequestration of PI(4,5)P2 in the inner plasma membrane leaflet [74]. Since PI(4,5)P2 and PI(3,5)P2 are involved in electrostatic interactions with proteins (e.g. MBP and MPZ) in plasma- and endolysosomal membranes, respectively, alterations in their spatial distribution and organization could lead to disturbances in myelination [74, 79]. Interestingly, due to the high affinity of sphingomyelin for cholesterol, redistribution of cholesterol across exo- and endoplasmic leaflets of the cell membrane is likely to occur, and possibly further contributes to the CMT1G demyelination [80]. In addition, PMP2 is required for efficient remyelination after nerve injury and regulates both ATP production and palmitate uptake in Schwann cells [72]. In conclusion, current evidence suggests that PMP2-related HPN are likely partially caused by sphingomyelin and PI homeostasis disruption, which influences other metabolic adaptations of Schwann cells.

##### CMT1D

CMT1D represents an autosomal dominant form of CMT caused by mutations in *early growth response 2* (*EGR2*) [74, 79]. EGR2 strongly stimulates the expression of several lipid metabolism-related enzymes, including choline kinase and stearoyl CoA desaturase [81]. In addition, EGR2 stimulates the expression of myelin proteins such as PMP22, PMP2, and MBP, all of which are recurrently implicated proteins in the spectrum of mutations causing demyelinating CMT [72, 81, 82]. *EGR2* mutations in CMT1D generally lead to a loss of function, resulting in impaired expression of genes involved in myelin protein (e.g. MAG, periaxin, PMP22, MPZ) and lipid metabolism (e.g. choline kinase, Stearyl coA desaturase, HMG CoA reductase) in Schwann cells [81]. Overall, whether these changes are cause or consequence remains unclear, but changes in phospholipid synthesis, fatty acid desaturation, and cholesterol synthesis are expected to disrupt overall myelin homeostasis, considering they are fundamental to PNS myelin [10].

##### MPZ-related Charcot-Marie-Tooth disease variants

Over 120 mutations in *MPZ* have been described to cause multiple forms of CMT (1B, 2I, 2J), and congenital hypomyelinating neuropathy 2 [83]. MPZ serves as a major integral membrane protein in PNS myelin, playing a crucial role in the homoadhesion of the myelin sheath wrapping process [84]. Several hypotheses have been proposed to understand the underlying pathological mechanisms associated with mutated MPZ in Schwann cells, with lipids emerging as key players in these processes. One such hypothesis suggests that MPZ undergoes electrostatic interactions with negatively charged lipids (PI, PG, phosphatidic acid [PA]) through its strongly positively charged cytoplasmic tail [85, 86]. In addition, MPZ canonically requires cholesterol for its exit from the endoplasmic reticulum to reach the myelin sheath [87]. Interestingly, a recent study by Plotkowski, et al. identified an interaction between the transmembrane regions of MPZ and PMP22, facilitating the formation of the MPZ-PMP22 complex in the plasma membrane [88]. Furthermore, the cytoplasmic tail of MPZ is necessary for correct tight membrane lipid packing, which is pivotal for myelin sheath formation [89]. In conclusion, lipid disturbances could significantly contribute to the underlying aetiology of peripheral neuropathies associated with *MPZ* mutations, but are currently underexplored.

##### CMT4

In addition to CMT1 forms, considerable evidence suggests that disturbances in lipid quantity, quality, and metabolism are triggers of demyelination in autosomal recessive variants of demyelinating forms of HPN, also known as CMT4. CMT4 disorders are characterized by defects in Schwann cell physiology and dysmyelination. To date, many different forms of CMT4 have been identified, including type B1, B2, D, E, J, which are associated with mutations in *MTMR2*,* MTMR13/SBF2*,* NDRG1*,* EGR2*, and *SAC3/Fig4*, respectively.

Mutations in the gene encoding myotubularin-related protein 2 (MTMR2) underpin CMT4B1, a severe HMSN with childhood onset that is characterized by myelin outfoldings and demyelination. MTMR2, a member of the myotubularin family, possesses a lipid phosphatase domain crucial for dephosphorylating PI(3)P and PI(3,5)P2 to form PI and PI(5)P, respectively. It is well-described to play an essential role in the myelination of peripheral nerves as reviewed in [90]. Berger, et al. showed that mutated MTMR2 results in loss of its phosphatase activity, reduced interaction with its regulatory binding partner MTMR13/SBF2, and lipid metabolic defects, impacting Akt levels in the PNS [91]. Furthermore, Sawade, et al. provided strong evidence that the Ras-related GTPase Rab35 associates with the myotubularin-related PI 3-phosphatases MTMR13 and MTMR2 to control myelination, modulating lipid-mediated mTORC1 activation, which is a critical regulator of myelin biogenesis [92]. In addition, MTMR2 coordinates mTORC1-dependent myelin synthesis and RhoA/myosin II-dependent cytoskeletal dynamics by regulating PI(3,5)P2 levels, thereby promoting myelin membrane expansion and longitudinal myelin growth [93]. Proof-of-concept experiments further showed that pharmacological inhibition of PI(3,5)P2 synthesis or mTORC1/RhoA signaling ameliorates CMT4B1 phenotypes.

CMT4 type J is a polyneuropathy caused by compound heterozygous mutations in *SAC3/Fig4* and is characterized by segmental demyelination and neurodegeneration [94]. SAC3, an evolutionary conserved phosphatase, stabilizes in the cytosol by interacting with the scaffold-associated regulator of PIKfyve (ArPIKfyve) [95]. SAC3 promotes the turnover of PI(3,5)P2 to PI(3)P and catalyzes PI(3,5)P2 synthesis by activating PAS complex (PIKfyve-ArPIKfyve-Sac3) [96]. Failure of this mechanism has been described to contribute to the pathogenesis of CMT4J, as patients show reduced stability in the PAS complex, rendering it more prone to degradation. Although the average levels of PI in CMT4J cohorts remain unchanged, there were high variations observed among individual patients [95]. Furthermore, in fibroblasts harbouring a *Fig4* mutation, a net decrease in PI(3,5)P2 levels was present [94]. For a subset of patients, aberrant endolysosomal vacuoles are apparent and correlate with reduced levels of PI(3,5)P2 and PI(3)P. This correlates with altered cellular PI levels affecting endosomal membrane fusion/fission and endocytic trafficking to lysosomes (reviewed in [97, 98]).

Finally, the N-myc downstream regulated gene (*NDRG1*)-inactivating mutations cause severe demyelination in CMT4D [99]. A few studies suggest that changes in the quantity and metabolism of lipids underpin cellular dysfunction in CMT4D pathology. For instance, mutations in *NDRG1* cause lipid metabolic defects in different breast cancer cell lines, markedly affecting fatty acid incorporation into neutral lipids and lipid droplets [100]. Moreover, NDRG1 regulates low-density lipoprotein receptor (LDLR) trafficking by regulating endosomal recycling and degradation in both epithelial A431 cells and murine oligodendrocytes, thereby affecting LDL-cholesterol uptake [101]. Studies in *NDRG*^*−/−*^ Alaskan Malamute dogs show decreases in SM, PE, and PC in peripheral nerves, suggesting impaired lipid processing and myelinating cell differentiation in CMT4D [99]. However, currently, the evidence for CMT4D describes lipid alterations more as a consequence of disturbed Schwann cell physiology than the true cause of the disease, although changes in PI species seem to be a common mechanism in multiple CMT4 variants. Therefore, further research is warranted to confirm these findings and to evaluate the relative contribution of these changes to CMT4D pathology.

In conclusion, there seem to be multiple different lipid species involved in Schwann cell-related CMT pathologies, but there are some visible trends present. CMT1G and multiple CMT4 variants (B1 and J) seem to have PI-substrates as a common denominator in their pathology. Furthermore, CMT1G may be indirectly related to PMP22-related pathologies through their shared disturbance of the cholesterol-sphingomyelin balance, an essential component in membrane asymmetry and important building blocks of PNS myelin. However, in order to really determine their aetiological similarities, more research is needed into these primary Schwann cell HPN.

### Lipid disturbances in axonal forms of HPN

**Fig. 2:**
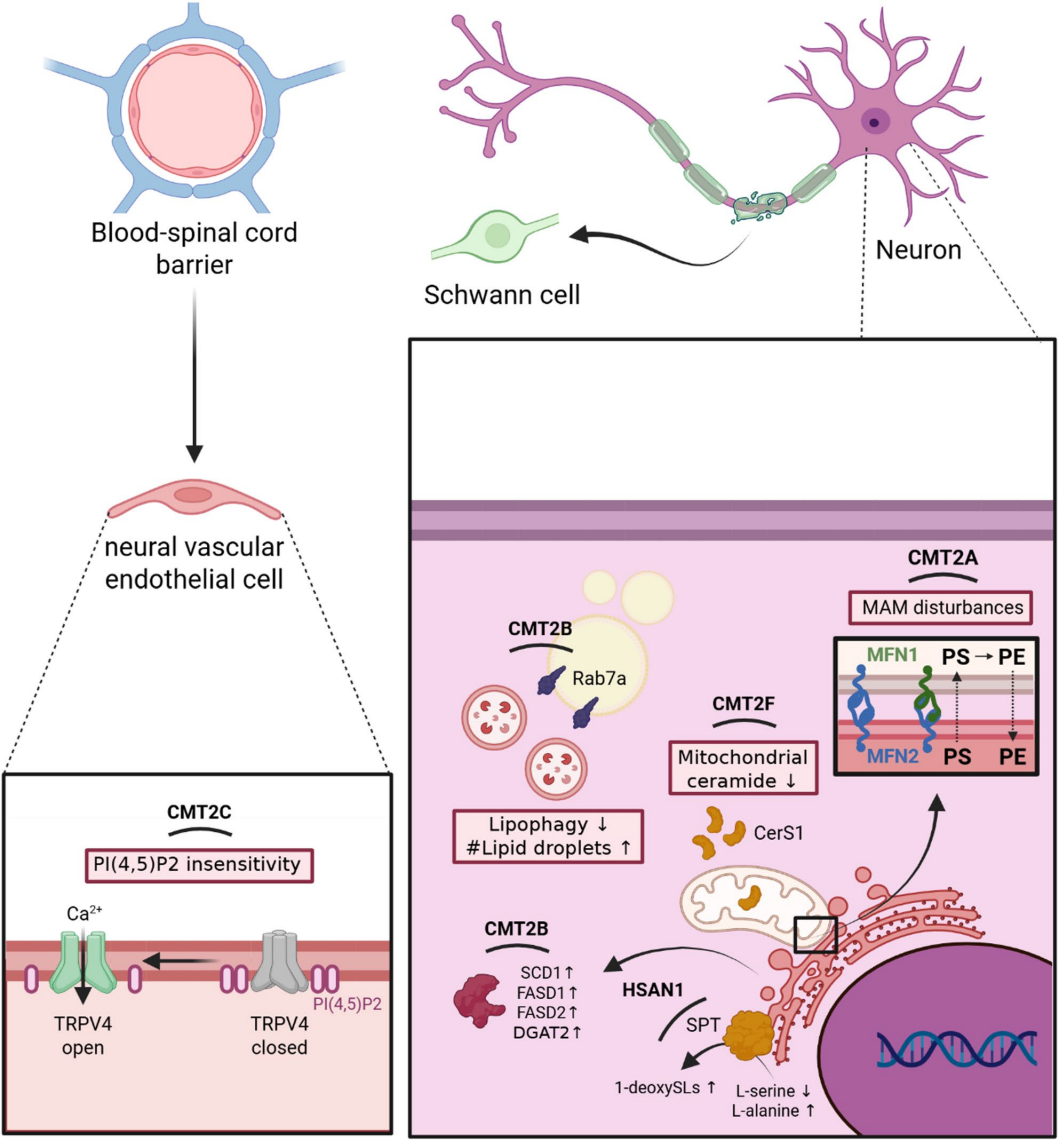
Graphical overview of lipid-related molecular perturbations in primary neuronal HPN. Disturbances in MAMs in CMT2A are caused by MFN2 mutations, leading to perturbed PS-PE conversion. CMT2B Rab7a mutations hamper lipophagy and result in an increased total number of lipid droplets. Furthermore, protein levels of SCD1, FADS1, FADS2, and DGAT2 are increased in CMT2B, influencing fatty acid saturation, elongation, and TG synthesis. CMT2C TRPV4 mutated variants are typically insensitive to PI(4,5)P2, usually resulting in uncontrolled channel opening and Ca^2+^ influx. However, despite common belief of CMT2C being a primary neuronal HPN, recent data shows an alternative origin in neural vascular endothelial cells. Reduced ceramide levels are found in CMT2F due to CerS1 disruption. HSAN1 mutations in SPT shift substrate usage from L-serine towards L-alanine, leading to increased formation of 1-deoxySLs. Despite many changes in lipid-related pathways being present in neuronal HPN, no common mechanism can be found among the different diseases. CMT: Charcot-Marie-Tooth disease; PI(P): phosphatidylinositol (phosphate); PS: phosphatidylserine; PE: phosphatidylethanolamine; HSAN1: hereditary sensory and autonomic neuropathy 1; MAM: mitochondria-associated membranes; TRPV4: transient receptor potential cation channel subfamily V member 4; MFN: mitofusin; Rab7a: Ras-related protein 7a; SPT: serine palmitoyltransferase; SCD1: stearoyl-CoA 9-desaturase; FADS: fatty acid desaturase; DGAT2: diacylglycerol O-acyltransferase 2

Given their extensive axonal projections and elevated metabolic needs, motor neurons and DRG sensory neurons are especially susceptible to genetic injuries, often resulting in axonal degeneration [102]. In addition to demyelinating CMT phenotypes, various forms of CMT2 predominantly arise from gene mutations that induce lipid changes in neurons, thereby disrupting their overall functionality. An overview of these disturbances in lipid homeostasis and lipid-related pathways is summarized in Table 2 and Fig. 2, respectively.

### Neuronal charcot-marie-tooth disease variants

#### CMT2A

The predominant form of primary neuronal HPN is CMT2A, caused by autosomal dominant mutations in the mitofusin-2 gene (*MFN2*) [103]. MFN2 resides in the outer mitochondrial membrane and plays a role in tethering mitochondria to specialized regions of the endoplasmic reticulum, known as mitochondrial-associated membranes (MAM) [104]. These interfaces preserve several essential functions. For example, the transport of PS from the MAM to mitochondria is facilitated from here, where the enzyme PS decarboxylase (PISD) converts it to PE [105]. Furthermore, MAMs regulate Ca^2+^ homeostasis, mitochondrial fusion and fission, and initiate autophagosome formation [106]. Loss of MFN2 or mutations in this gene have several consequences. Most CMT2A-associated MFN2 mutations cause mitochondrial dysfunction and fragmentation, and mitochondrial aggregation in Mfn2^H165R/H165R^ cells [107]. In addition, heightened apoptotic responses occur due to elevated ER stress [108]. Disruption of MAM-mitochondria associations also affects phospholipid composition, changing the subpopulations of PE and PC in terms of fatty acyl chain length and degree of unsaturation [109]. In MFN2 KO mouse embryonic fibroblasts (MEFs) subjected to ethanolamine deprivation, total PE levels decreased, with a significant decline in the PISD-driven PE subpopulation, comprising PE species with four or more unsaturated bonds. Conversely, an opposite trend was noted for PE species synthesized via the CDP-ethanolamine pathway, characterized by three or fewer unsaturated bonds [109]. Together, these findings highlight that MFN2-mediated neuropathology may, at least in part, arise directly from PE synthesis, alongside impairments in Ca^2+^ homeostasis, autophagy and mitochondrial dynamics.

#### CMT2B

CMT2B is an autosomal dominant HPN caused by missense mutations in the *RAB7* gene, a member of the family of small GTPases known to regulate microtubule trafficking of lysosomes, late endosomes, and autophagosomes within axons [110]. Additionally, RAB7 serves as a conserved component within lipid droplet membranes and is suggested to contribute to the autophagic degradation of these lipid droplets and lipid metabolic signaling [111]. Giudetti, et al. revealed profound differences in lipid metabolism and lipid droplet accumulation in CMT2B patient-derived fibroblasts compared to healthy donor cells. CMT2B patient-derived fibroblasts further showed elevated protein expression levels of Δ-9 desaturase (SCD1), as well as fatty acid desaturase 1 (FADS1) and 2 (FADS2), compared to healthy control cells [112]. SCD1 plays a pivotal role in the conversion of palmitic acid (16:0) and stearic acid (18:0) into palmitoleic acid (16:1) and oleic acid (18:1), respectively, with the latter serving as the principal substrate for TG synthesis [113]. Correspondingly, CMT2B patient-derived fibroblasts showed increased monounsaturated fatty acid levels, particularly oleic acid (C18:1), compared to healthy control cells. These cells also exhibited an increased percentage of arachidonic acid (C20:4, n-6) and eicosapentaenoic acid (C20:5, n-3), accompanied by a concurrent reduction in the percentage of linoleic acid (C18:2, n-6). Moreover, CMT2B-derived fibroblasts showed elevated expression levels of *de novo* lipogenic enzymes, acetyl-CoA carboxylase (ACC) and fatty acid synthase (FAS), along with an increased rate of labeled acetate incorporation into fatty acids. CMT2B fibroblasts also showed a higher expression of DGAT2, an enzyme pivotal in TG synthesis primarily utilizing *de novo*-synthesized fatty acids, leading to the accumulation of more TG in the form of lipid droplets compared to healthy controls. In addition, an elevated cholesterol ester/cholesterol ratio was observed in CMT2B-derived fibroblasts. The increased tendency for lipid droplet accumulation in CMT2B-derived fibroblasts is further supported since TG and cholesterol esters are the primary neutral lipids comprising the core of lipid droplets [114]. These substantial alterations in lipid metabolism were ascribed to perturbations in the processing of SREBP-1. This transcription factor governs a broad spectrum of genes involved in lipid biosynthesis, encompassing *de novo* lipogenesis (ACC1, ACC2, and FAS), TG synthesis (DGAT2), fatty acid desaturation (SCD1, FADS1, and 2), and fatty acid elongation [112]. Despite alterations in lipid metabolites and enzymes being prominent in CMT2B models, they seem to be indirect effects of the disease. However, it remains to be investigated whether modulation of these pathways can rescue the CMT2B pathobiology.

#### CMT2C

Autosomal dominant mutations in the ankyrin domain (ARD) of TRPV4 contribute to several channelopathies, including CMT2C [115]. Direct interaction between TRPV4 and the inositol head group of PI(4,5)P2 is known to be important for ion channel activity regulation, despite significant controversy concerning their interaction domains [116]. Hydrolysis of membrane PI(4,5)P2 has been associated with increased TRPV4 channel activities and cell death. Additionally, disease-associated TRPV4 mutations abolished PI(4,5)P2 binding and sensitivity, resulting in increased TRPV4 activities and elevated cell death [117]. Despite common belief that CMT2C has a primary neuronal origin, emerging data indicate that TRPV4-mediated neuropathies are driven, at least in part, by non-cell-autonomous mechanisms. Recent work from the Sumner Lab has provided strong evidence that gain-of-function TRPV4 mutations in the ARD increase Ca^2+^ influx leading to dysfunction and death of neural vascular endothelial cells at the blood-spinal cord barrier, leading to perturbed barrier integrity [118]. When investigating this mutation further, it was found that this TRPV4-R269C mutation likely influences PI(4,5)P2 binding properties of the ion channel, leading to overactivation [119]. Furthermore, by means of selective deletion of the mutant allele in the cell types comprising the neuromuscular unit, no direct rescue effect was found when performed in Schwann cells or neurons [118]. Currently, PI(4,5)P2 involvement in TRPV4 neural vascular endothelial cell dysfunction in CMT2C is one of the proposed disturbed molecular mechanisms, making investigation of PI(4,5)P2 to contribution of the disease particularly relevant.

#### CMT2F

CMT2F is an autosomal dominant HPN caused by mutations in the *HSPB1*-gene encoding heat shock protein 27 (Hsp27), which has been found to co-localize with ceramide synthase (CerS), the enzyme responsible for catalyzing the generation of the sphingolipid ceramide [120, 121]. Hsp27 KO nerves showed decreased Cer levels, particularly in longer chain-length Cer (C24 and C26) with saturated acyl chains. This decrease in Cer occurs acutely in the mitochondria in CMT2F mutant cells, which presented with less mitochondrial colocalization of the isoform CerS1 [120]. Data on lipid metabolism changes in CMT2F are currently limited to a single study, warranting further investigation into the role of CerS in the pathology of CMT2F. Currently, therapeutic approaches for CMT2F are more directed towards the rescue of mitochondrial function and axonal transport by means of HDAC6 inhibition, which may indirectly restore some lipid changes seen in Hsp27 KO nerves [122].

In conclusion, the current literature on lipid disturbances in CMT2 highlights pivotal roles for correct mitochondrial functioning, which relies on MAM formation and mitochondrial stability, and fatty acid synthetic pathways in the establishment of functioning peripheral nerve axon bundles. However, the evidence suggests that in CMT2 variants, these lipid alterations are a consequence of the disease and likely only partly contribute to the pathophysiology of these HPN.

### Other primary neuronal HPN

#### Hereditary sensory and autonomic neuropathy

Beyond axonal forms of CMT, lipid disturbances are apparent in other peripheral axonal diseases. In the HSAN group, which primarily affects sensory and autonomic neurons, the disease leads to progressive degeneration and dying back of the axons of DRG neurons. HSAN1, an autosomal dominant axonal neuropathy, arises from missense mutations in the *SPTLC1* or *SPTLC2* gene, which encodes two subunits of serine-palmitoyltransferase (SPT). SPT catalyzes the first and rate-limiting step in the *de novo* sphingolipid synthesis [40, 49]. Mutant SPT variants demonstrate a shift from their typical substrate L-serine to L-alanine, resulting in the accumulation of 1-deoxysphingolipids (1-deoxySLs), which are atypical bioactive sphingolipids in neurons [123–125].

Of interest, a 10% L-serine-enriched diet has shown a reduction of 1-deoxySL levels in C133W SPTLC1 mutant mice, a reliable mouse model for HSAN1 [124]. In support of the detrimental impact of 1-deoxySL, overexpression of wild-type SPTLC1 in mice led to a decrease in 1-deoxySL levels, and a reduction of symptoms associated with HSAN1 [126]. A study using HSAN1 patient-derived iPSC-sensory neurons further demonstrated that missense mutations in *SPTLC1* and *SPTLC2* lead to reduced GM1a, GD1b, GT1b, and glycosphingolipid levels, contributing to impaired neuronal branching, paranodal defects, and internode fragmentation, potentially hindering axo-glial interactions [127]. Here, L-serine supplementation counteracted the reduced ganglioside and glycosphingolipid levels [127]. In a 10-week human pilot study with HSAN1 patients, treatment with 200 and 400 mg/kg/day of L-serine resulted in a significant decrease of 1-deoxySL levels by 2- and 4-fold, respectively. Additionally, this treatment led to improved skin resilience and enhanced hair/nail growth, although it did not affect sensory perception [124]. Currently, HSAN1 patients are being recruited for a 12-month follow-up study (SENSE trial) that will investigate the influence of L-serine supplementation on a larger scale (additional information: clinicaltrials.gov; study-ID: NCT06113055) [124]. Collectively, these findings underscore the role of 1-deoxySLs in the development of HSAN1 and suggest the potential efficacy of L-serine supplementation as a therapeutic approach for HSAN1 [123].

Extensive evidence indicates that 1-deoxySL adversely affects the physiology of multiple cell types, manifesting its effects in various functionally distinct manners. For instance, exposure of primary mammalian neurons to deoxysphingoid bases results in dose- and time-dependent neurotoxicity. This is characterized by abnormal handling of Ca^2+^ by both the endoplasmic reticulum and mitochondria, along with a decline in mitochondrial membrane potential [128]. In a recent study, exposure to an alkyne analog of 1-deoxysphinganine (doxSA), the metabolic precursor of deoxySLs, amplified mitochondrial swelling in DRG neurons [129]. Additionally, it induced mitochondrial fragmentation and endoplasmic reticulum stress in MEFs, resulting in cellular dysfunction and toxicity. Interestingly, pharmacological inhibition of CerS partially mitigated these effects, suggesting a potential therapeutic strategy [129]. Furthermore, 1-deoxySAs impede the migration of fibroblasts in a dose- and time-dependent manner due to their conversion into 1-deoxy-sphingosines [130]. Inhibition of downstream metabolism by hampering N-acetylation showed promise in improving perturbed migration induced by 1-deoxySAs [130], which holds potential for enhancing wound healing in HSAN1 patients. Marshall, et al. observed a notable increase in lipid droplet abundance in lymphoblasts from HSAN1 patients expressing mutant SPTLC1 proteins, suggesting a connection between elevated lipid droplet levels and disturbed cellular function [131]. Also, 1-deoxySLs were found to induce an accumulation of autophagosomes and lysosomes in macrophages, reflecting an increased autophagic flux, promoting the formation of crystals and activating the NLRP3 inflammasome [132]. Finally, a recent study showed that the impaired lateral segregation of 1-deoxySLs into ordered domains within a fluid membrane may stem from the configuration of the spingoid base double bond rather than the structure of its C1 functional group. These alterations potentially contribute, at least in part, to the pathophysiological effects of 1-deoxySLs [133]. All in all, these findings indicate that 1-deoxySLs impact mitochondrial and ER function, plasma membrane fluidity, and autophagy.

Notably, mutations in SPTLC1 can also cause forms of amyotrophic lateral sclerosis (ALS), depending on the protein domain that is affected. ALS *SPLTC1* mutations are typically restricted to the first transmembrane domain, which is sensitive to ceramide and ORMDL protein homeostatic feedback mechanisms, whereas HSAN1 mutations are found in multiple other protein domains. The ALS *SPTLC1* mutations result in an increased production of sphingolipids, ceramides, and glucosylceramide in particular, leading to perturbations in motor neuron function [134]. Therefore, L-serine supplementation treatments may not be suitable for these ALS cases, as they could potentially exacerbate the production of cytotoxic sphingolipids [134].

## Novel approaches and model systems in lipidomic research

One main shortcoming of the current lipid metabolism research field is the techniques employed in experiments. Since it is still challenging to provide comprehensive overviews of lipid species changes and possible intramolecular alterations in lipids, it is important to discuss the current state-of-the-art methodology that could be used in future studies. In order to investigate lipid class abundances and composition, soft ionization mass spectrometry (MS) techniques are typically used. Most commonly, researchers employ electrospray ionization (ESI), desorption electrospray ionization (DESI), and matrix-assisted laser desorption/ionization (MALDI), with the latter providing a method that can reveal the spatial distribution of lipid species [135–137]. These MS techniques already offer major pieces of information on lipidomic disturbances in HPNs at an unprecedented level of detail [52, 53, 113, 114]. While significant strides have been made in the field of lipidomics, there are still several challenges to address, particularly concerning the analysis of subcellular alterations in lipid species. With respect to the latter, MS imaging is hindered by its spatial resolution, which currently can reach approximately 6 μm [138], and organelle isolation protocols are often time-consuming or lack efficiency. Interestingly, a methodology that would be useful in overcoming this limitation is Raman microscopy, which can achieve a spatial resolution between 200 and 500 nm depending on the microscope system. Raman spectra can further provide information on physicochemical properties, viscosity, and (sub)cellular distribution of lipid classes [139, 140]. Furthermore, different improved methods of Raman microscopy, like Coherent Anti-stokes Scattering (CARS) and Stimulated Raman Scattering (SRS) microscopy, provide enhanced Raman signal acquisition, which makes analyses of cell and tissue spectra more feasible [141]. Combined Raman-MS imaging pipelines, therefore, could provide novel pathophysiological changes in many HPNs [142], with the possibility to give insights into subcellular lipid trafficking, lipid profile discrepancies within cell-types (e.g. Schwann cells that myelinate sensory and motor neurons).

Alongside spatial resolution improvements, alternative methods for MS have been developed that could provide essential information in understanding the pathophysiology of HPN. By means of isotope tracer administration to biological samples, some research groups have been able to implement MALDI-MS to determine the relative formation of lipid metabolism pathway intermediates and end-products [143, 144]. Furthermore, by implementing ozone-induced dissociation on a mobility-enabled quadrupole time-of-flight MALDI-MS system, (phospho)lipid isomers were detectable and distinguishable [145]. Consequently, *sn-1* and *sn-2* fatty acid discrimination and fatty acid chain double bond location determination can be achieved. These findings might hold promise in the use of specific lipases for therapeutic purposes, as well as dietary interventions.

Aside from exact identification of lipid species, lipid-related idiosyncrasies like fluidity, polarity, rigidity, and packing are equally important parameters in the maintenance of cell membrane integrity. Amphiphilic fluorescent probes — like Laurdan, Di-4-ANEPPDHQ, and Nile Red, which are most commonly used — are such a lipidomic tool that not only permits spatial polarity analyses, but even extension into temporospatial lipid dynamics and ordering into lipid domains. Furthermore, the development of novel imaging probes, such as NR4A and pro12A, has substantially advanced plasma membrane analysis by enabling outer leaflet specificity or exhibiting reduced plasma membrane affinity. These properties facilitate nanoscale investigations through Point Accumulation for Imaging in Nanoscale Topography (PAINT) super-resolution microscopy, thereby providing important opportunities to elucidate PMP22- and PMP2-associated pathomechanisms linked to cholesterol and sphingomyelin leaflet asymmetry. The methodological advances underlying these approaches are comprehensively detailed by Collot, et al. [146]. In addition, computational modeling offers a powerful complementary tool to further delineate the impact of altered lipid species abundance on membrane organization and dynamics [147].

Currently, polarity and applied computational methods are underused, especially in lipidomic approaches in PNS research, and many applications of polarity probes remain to be implemented in the PNS field. In conclusion, although a basis of knowledge has been established in the last few years, there is potential for improvement in PNS lipid research by employing newly developed methods. A final important note is that lipidomic research in peripheral neuropathies must carefully consider the experimental context, as studies on cell, tissue, and organism models (in vitro, ex vivo, in vivo) provide distinct insights. In particular, Schwann cells differ markedly between in vivo myelinating states and non-myelinating 2D cultures, which can profoundly impact lipid profiles and interpretation.

## Concluding remarks

Currently, a prevailing trend in lipid disturbances has been observed across the spectrum of (HPN). Not only are demyelinating diseases associated with Schwann cells affected, but neuronal variants also appear to be susceptible to lipid alterations. Interestingly, these changes do not seem to be limited to certain pathways, but do lead to perturbations within the same lipid species. Demyelinating HPN primarily exhibit susceptibility to disturbances in cholesterol, phospholipid, and phosphoinositide species, whereas neuronal HPN are generally affected by disruptions in sphingolipids, and certain phospholipids. However, it should be noted that fully distinguishing the causes and consequences of the disease is not possible. For demyelinating variants, the above-described lipid species all seem heavily involved in myelination, either through pathway activation, membrane leaflet asymmetry, or general provision of lipid building blocks to generate enough myelin sheath layers. Interestingly, cholesterol shortage in the membrane and trafficking thereof seems to underpin PMP22-related HPN, while CMT1G, CMT4B and CMT4J all are hindered through reduced and/or disproportional synthesis or membrane distribution of PI species. Therefore, targeting these common pathways seems interesting to pursue in future studies, alongside increasing the overall content of myelin sheath lipids. Currently, no hereditary peripheral neuropathies (HPN) have been clearly linked to lipid metabolism–related defects in non-myelinating Schwann cells, and their contribution to disease pathogenesis remains unestablished. By contrast, lipid alterations in neurons are more heterogeneous, comprising both primary pathogenic changes, such as sphingolipid dysregulation in HSAN, and secondary changes affecting phospholipids and fatty acids in CMT2. Nevertheless, it seems that neurons in the PNS are primarily susceptible to 1-deoxysphingolipid imbalances, hindering membrane integrity and/or autophagolysosomal flux, leading to cellular toxicity. One explanation for the cellular discrepancy is the role of these lipid species, since Schwann cell-related HPN are mainly affected by species that are essential for correct myelination, while changes in the lipidome in neuronal HPN are associated with more nuanced intracellular housekeeping functions and typically seem less pronounced contributors to disease pathobiology. Further research is needed to distinguish whether these changes in lipid species are main contributors or side effects of the pathophysiology of specific HPN. Furthermore, due to technical and experimental limitations, certain avenues in lipid metabolism research may be under-investigated and might contribute to a degree of availability bias.

Insights into perturbed lipid metabolism in HPN will likely aid in developing therapies for this diverse field that currently lacks definitive answers. First, it seems that primary neuronal HPN lack any common lipid mechanism, and therefore targeting multiple neuronal HPN through a common mechanism does not seem feasible. However, on the contrary, primary Schwann cell-related HPN seem to have 3 main causes, namely hampered cholesterol trafficking/membrane content, lipid storage defects and PI-related species imbalances. Additionally, in broader terms, reduced lipid biosynthesis/turnover seems to be an additional commonality among dysmyelinating HPN. Therefore, by modulating metabolic pathways or through lipid-based dietary interventions, it may be possible to treat multiple Schwann cell–related HPN, representing a promising avenue for future investigation. Currently, multiple clinical studies are already ongoing for these diseases. While these trials do not specifically target lipid metabolism, evaluating lipid profiles in patients may provide insights into primary and secondary effects of disease and could inform future therapeutic strategies. For instance, a phase III trial for CMT1A using a low-dose combination pill of baclofen, naltrexone and D-sorbitol (PXT3003) designed to reduce PMP22-overexpression is being re-evaluated in a second phase III trial (NCT04762758). Additionally, for CMT1A, 1B and 1E, a phase I trial using IFB-088 is being conducted, aiming to improve UPR and ER stress and thus preventing protein misfolding [148]. Furthermore, In the broader category of axonal CMTs such as CMT2A and other CMT2 types, a symptomatic small-molecule (NMD670) aimed at improving neuromuscular transmission is under Phase 2a testing in adults with genetically confirmed CMT1 or CMT2 (NCT06482437). For both CMT1 and CMT2, multiple phase 1 trials are ongoing using AGT-100,216 to inhibit HDAC6 [149]. Interestingly, despite uncertainty in its contribution to the aetiology, CMT2A phospholipid and sphingolipid changes are being explored as biomarkers as well (NCT04881201). For CMT2C, a first-in-human Phase I trial of ABS-0871 (a TRPV4 inhibitor) has recently been initiated in healthy volunteers (with subsequent plans for CMT2C patients). As mentioned above, HSAN1 patient recruitment for the SENSE trial is currently ongoing, which will investigate the influence of L-serine supplementation (NCT06113055). For a full, detailed list of all ongoing CMT clinical trials, we kindly refer to the review by De Grado, et al. [150]. Despite currently not being a target of interest in clinical studies, there is the strong involvement of lipid metabolism in many HPN, directly or indirectly, and general peripheral nerve lipid homeostasis makes their rescue a promising therapeutic avenue to explore in the upcoming years.

### Future perspectives

Despite all evidence provided for a general role for lipids in HPN and some commonalities between different HPN, further specification of these lipid perturbations will be essential for the advent of novel drug therapies and lipid diets as treatment options. Alongside basic quantitative lipid species analyses, details regarding subspecies, spatial distribution of lipids in peripheral nerve tissues and cells, and information regarding fatty acid chemical composition and placement in lipid species (e.g. *sn-1* or *sn-2* positions in phospholipids), will be essential to comprehend the different aspects of HPN. Furthermore, the activity of major lipid anabolic and catabolic enzymes remains ill-defined and could shed light on pathobiological mechanisms that are missed by quantitative transcriptomic and lipidomic approaches. By employing the methodology described above and considering the literature on lipids in HPN, researchers will gain access to new avenues for modulating lipid metabolism in HPN.

**Table 1:** Summary of lipid alterations in primary Schwann cell HPN

Associated gene (OMIM)	Protein name & Gene function (OMIM)	Disease subtype (OMIM)	Mode of inheritance	Lipidomic alteration
PMP22 (601097)	Peripheral myelin protein 22; major component of peripheral myelin, involved in Schwann cell growth and differentiation	CMT1A (118220)	AD	CMT1A rat SN myelin [52]: • cholesterol, PC, PE P-, SM, cerebroside, HexCer, ceramide, GalCer (vs. protein content myelin) • PE-P decrease and TAG increase (vs. lipid content myelin) CMT1A rat SN [53]: • PC, PS, SM (long, saturated C chains), PI, DG, HexCer decrease • PE (42:1) increase C3-PMP22 SN [54]: • cholesterol, TG (US), SM, HexCer, dhCer and Hex2Cer decrease • general PL decrease • saturated long chain PC increase iPSC-SCP [54]: • cholesterol, SM, sphingolipids, short-chain PL decrease • shorter acyl-chain PS & longer-acyl chain (poly)unsaturated PC, PI, PG, PE, PE O-, and PE P- increase wild-type-PMP22 overexpressing rat Schwann cells [58]: • cytosolic cholesterol increase (lysosomal disposition) • membrane cholesterol decrease
		HNPP (118220)	AD	pmp22-KO mouse & pmp22 shRNA KD rat Schwann cells [55]: • cholesterol increase in perinuclear region • cholesterol decrease in plasma membrane
		CMT1E (118300) DSS/CMT3 (145900)	AD AD, AR	TrJ mouse model [58]: • perinuclear entrapment of cholesterol in Golgi apparatus • halved cholesterol levels (+/-|-/-) Tr mouse model [66, 67]: • PC & PI decrease • lower polar lipid/absolute lipid content • relative abundance PC & SM is constant over time (decrease in wild-type) • PC, PI & cardiolipin is more abundant (compared to total polar lipids) • decreased palmitate levels
PMP2 (170715)	Peripheral myelin protein 2; Involved in lipid dynamics and myelin membrane stability	CMT1G (618279)	AD	PMP2-mutated HeLa cells [74]: • PI(4,5)P2 sequestration • increased transbilayer movement of SM to inner leaflet PMP2-KO HeLa cells: • SM increase in outer layer PMP2-overexpression: • SM decrease in outer layer
EGR2 (129010)	Early growth response 2; a transcription factor that is a prime regulator of Schwann cell myelination.	CMT1D (607678)	AD	/
MPZ (159440)	Myelin protein zero; Myelin protein-zero is the major structural protein of peripheral myelin.	CMT1B (118200)	AD	/
MTMR2 (603557)	Myotubularin-related protein 2; a protein that belongs to the myotubularin family, which is characterized by the presence of a phosphatase domain	CMT4B1 (601382)	AR	/
Figure 4 (609390)	Phosphoinositide 5-phosphatase; tight regulator of PI(3,5)P2, involved in endosomal membrane dynamics	CMT4J (611228)	AR	Subset of CMT4J patient fibroblasts [95]: • reduced levels of PI(3,5)P2 and PI3P
NDRG1 (605262)	N-Myc Downstream Regulated 1; signaling protein involved in cell differentiation and growth arrest	CMT4D (601455)	AR	NDRG1 -/- Alaskan Malamute dog peripheral nerves [99]: • strong SM decrease, and subtle decreases in PC and PE

PMP22: peripheral myelin protein 22; PMP2: myelin P2 protein; NDRG1: N-myc downstream-regulated gene 1 protein; CMT: Charcot-Marie-Tooth disease; HNPP: hereditary neuropathy with liability to pressure palsies; DSS: Dejerine-Sottas syndrome; AD: autosomal dominant; AR: autosomal recessive; SN: sciatic nerve; iPSC-SCP: induced pluripotent stem cell-Schwann cell precursor; PC: phosphatidylcholine; PE (P-): (plasmalogen-) phosphatidylethanolamine; PI(P): phosphatidylinositol (phosphate); SM: sphingomyelin; HexCer: hexosylceramide; GalCer: galactosylceramide; Tr(J): Trembler(-J); shRNA: short hairpin RNA; KD: knockdown; wt: wild-type

**Table 2:** Summary of lipid alterations in primary axonal HPN

Associated gene (OMIM)	Full name & Gene function (OMIM)	Disease subtype (OMIM)	Mode of inheritance	Lipidomic alteration
MFN2 (608507)	Mitofusin 2; involved in mitochondrial dynamics and MAM organization	CMT2A (609260)	AD	MFN2-KO MEFs [109]: • PE and PC FA population alterations o decrease in PE with > 3 unsaturated bonds o increase in saturated PE & PE with 1–3 unsaturated bonds
RAB7 (602298)	Ras-associated protein RAB7; small GTPase, involved in endocytic and autophagic vesicle fusion, vesicle trafficking, and vesicle maturation	CMT2B (600882)	AD	CMT2B patient-derived fibroblasts [114]: • increased MUFA abundance (mostly oleic acid [C18:1]) • increase in arachidonic acid (C20:4, n-6) and eicosapentaenoic acid (C20:5, n-3) • reduction in linoleic acid (C18:2, n-6) • more labeled acetate incorporation into FA • elevated cholesteryl ester/cholesterol ratio
TRPV4 (605427)	Transient receptor potential cation channel, subfamily V, member 4; cation channel that mediates calcium influx in response to physical, chemical, and hormonal stimuli	CMT2C (606071)	AD	/
HSPB1 (602195)	Heat-shock 27-KDa protein 1; ubiquitin-binding protein involved in proteasomal degradation	CMT2F (606595)	AD	Hsp27 KO mouse SN [120]: • decreased ceramide levels (mostly saturated long chain ceramides C24 and C26)
SPTLC1 (605712) SPTLC2 (605713)	Serine palmitoyltransferase, long-chain base subunit 1/2; subunit of the rate-limiting enzyme in sphingolipid biosynthesis	HSAN1A (162400) HSAN1C (613640)	AD	mutated SPTLC1 [125]: • increased formation of 1-deoxysphingolipids

MFN2: mitofusin-2; RAB7: Ras-related protein Rab-7a; HSPB1: heat shock protein beta-1; SPTLC: serine palmitoyltransferase; CMT: Charcot-Marie-Tooth disease; HSAN: hereditary sensory and autonomic neuropathy; AD: autosomal dominant; AR: autosomal recessive; PC: phosphatidylcholine; PE: phosphatidylethanolamine; (MU)FA: monounsaturated fatty acid; SN: sciatic nerve; iPSC: induced pluripotent stem cell; HDL: high-density-lipoprotein; MAM: mitochondria-associated membrane

## Data Availability

Not applicable.

## Abbreviations

1-deoxySL: 1-deoxysphingolipids
ABCA1: ATP-binding cassette transporter 1
ACC: Acetyl-CoA carboxylase
acDase: Acid ceramidase
AD: Autosomal dominant
ApoE: Apolipoprotein E
AR: Autosomal recessive
ARD: Ankyrin domain
CARS: Coherent Anti-stokes Scattering
Cer: Ceramide
CerS: Ceramide Synthase
CMT: Charcot-Marie-Tooth disease
deoxyCer: Deoxyceramide
deoxydhCer: Deoxydihydroceramide
DESI: Desorption electrospray ionization
DG: Diglycerides
dhCer: Dihydroxyceramide
dHMN: Distal hereditary motor neuropathy
DSS: Dejerine-Sottas Syndrome
EGR2: Early growth response 2
ESI: Electrospray ionization
FADS: Fatty acid desaturase
FAS: Fatty acid synthase
GALC: Galactosylceramidase
GalCer: Galactosylceramide
GJB1: Gap junction beta 1
HDL: High density lipoprotein
Hex2Cer: Dihexosylceramide
HexCer: Hexosylceramide
HMSN: Hereditary motor and sensory neuropathy
HPN: Hereditary peripheral neuropathies
HSAN: Hereditary sensory and autonomic neuropathy
Hsp27: Heat shock protein 27
iPSC-SCP: Induced pluripotent stem cell-Schwann cell precursor
LDLR: Low-density lipoprotein receptor 1
MALDI: Matrix-assisted laser desorption/ionization
MAM: Mitochondrial-associated membranes
MEF: Mouse embryonic fibroblast
MFN2: Mitofusin-2
MPZ: Myelin protein zero
MS: Soft ionization mass spectrometry
PA: Phosphatidic acid
PAINT: Point Accumulation for Imaging in Nanoscale Topography
PC O-: Ether-linked phosphatidylcholine
PC P-: Phosphatidylcholine plasmalogens
PE: Phosphatidylethanolamine
PE O-: Ether-linked phosphatidylethanolamine
PE P-: Phosphatidylethanolamine plasmalogens
PG: Phosphatidylglycerol
PISD: Phosphatidylserine decarboxylase
PMP2: Myelin P2 protein
PMP22: Peripheral myelin protein 22
PNS: Peripheral nervous system
PS: Phosphatidylserine
PTEN: Phosphatase and tensin homolog
SCD1: Δ-9 desaturase
SGMS1: Sphingomyelin synthase 1
SL: Sphingolipid
SM: Sphingomyelin
SN: Sciatic nerve
SPT: Serine-palmitoyltransferase
SRS: Stimulated Raman-Scattering
TG: Triglycerides
Tr: Trembler
Trj: Trembler-J
